# Estimation of serum matrix metalloproteinases among patients of oral squamous cell carcinoma

**DOI:** 10.12669/pjms.35.1.68

**Published:** 2019

**Authors:** Nakhshab Choudhry, Sana Sarmad, Noor ul Ain Waheed, Aamir Jamal Gondal

**Affiliations:** 1 Prof. Dr. Nakhshab Choudhry, PhD. Department of Biochemistry, King Edward Medical University, Lahore, Pakistan; 2 Dr. Sana Sarmad, MPhil. Department of Biochemistry, Rashid Latif Medical College, Lahore, Pakistan; 3 Dr. Noor ul Ain Waheed, MPhil. Department of Biochemistry, King Edward Medical University, Lahore, Pakistan; 4 Mr. Aamir Jamal Gondal, MPhil. Department of Biomedical Sciences, King Edward Medical University, Lahore, Pakistan

**Keywords:** Squamous cell carcinoma, Matrix metalloproteinase, Enzyme-linked immunosorbent assay

## Abstract

**Objective:**

To estimate the serum levels of matrix metalloproteinases in oral squamous cell carcinoma patients and in healthy subjects.

**Methods:**

In this observational study, biopsy diagnosed oral squamous cell carcinoma patients (n= 38) were recruited from Mayo Hospital, Lahore during 2016 to 2017. Age and gender matched Controls (n= 38) were also included. Venous blood sample of each participant was drawn, serum separated and the levels of matrix metalloproteinases were measured by multiplex ELISA.

**Results:**

Serum levels of MMP-1, -8, -10, -12 and -13 in OSCC patients showed statistically significant increase as compared to control group (p < 0.01). The MMP-12 predicted the presence of OSCC with highest AUC of 0.836 (95% CI [0.733 to 0.911]) for sensitivity and specificity of 80% and 78.9%, respectively for a cut-off value of 16.13 pg/ml.

**Conclusions:**

MMP-12 has been found to have significant sensitivity and specificity to qualify as a diagnostic biomarker.

## INTRODUCTION

Oral Squamous Cell Carcinoma (OSCC) has the highest incidence among oral cancers globally. Despite recent advances in OSCC therapy, its 5-year survival rate is only as high as 55%.^1^ Multiple risk factors have been linked with OSCC, including such specific factors as tobacco- and alcohol-use (substance-use). Regardless of this strong association, a considerable fraction of patients develop OSCC without exposure to them, underlining the role of genetic predisposition and oncogenic viruses.^2^

Matrix metalloproteinases (MMPs) are special class of proteases that are categorized in five groups as per their substrate specificity: collagenases (MMP-1, -8 and -13), gelatinases (MMP-2 and -9), stromelysins (MMP-3, -10, and -11), matrilysins (MMP-7) and membrane-type (MMP-14 to -17, -24 & -25). More than 25 distinct MMP genes have been identified in humans to date.^3^

Generally, there is a relationship between proteases level and cancer dissemination. Increased expression of MMPs such as MMP-1, -2, -3, -7, -9, -13, -14 are associated with cancer progression, i.e. poor prognosis, poor cancer cell differentiation, invasive cancer stage with low patient survival and metastatic spread to distant sites.^4^

Potential of OSCC to metastasize rests on its ability to break through the basement membrane, to penetrate the surrounding tissues and vascular structures and to bring about new vessel formation for tumor dissemination. Invasive potential of OSCC is positively attributed to the ability of neoplastic cells to deploy MMPs that are synthesized by the patient’s stromal cells. Many recent studies have reported the impact of MMPs in the advancement of OSCC either directly altering the extracellular environment or indirectly through initiation of vascular regression.^5^,^6^ Currently employed clinical tools for the detection of OSCC like biopsy, exfoliative cytology and vital tissue staining are generally applicable to a select group of patients and have distinct limitations.^7^

Little data are available for a biomarker that could aid in the detection of primary OSCC at non-detectable cancer tissue or precursor stage. Certain biomarkers may serve threefold advantage; for detection at an earlier stage, as prognostic marker, and a therapeutic target.^8^ MMPs are one of those potential markers that can theoretically serve all of these purposes. Determination of MMP levels in various tissue fluids has been suggested as easy, quick and noninvasive tool for primary diagnosis and subsequent prognostic monitoring of diseases.^9^

Some previously published studies indicated that certain multiplex panels of biomarkers have significant (85%) diagnostic accuracy in small study cohorts of OSCC patients.^10^ Many of these studies concluded that a wider analytic panel would contribute significantly to clinical benefits. However, early diagnosis may warrant avenues for better clinical management.

No study is currently available in Pakistani population concerning the measurement of serum MMPs levels. In present study, we measured the levels of MMP-1, -2, -3, -7, -8, -9, -10, -12, and -13 in OSCC patients and healthy subjects.

## METHODS

In this observational study thirty-eight biopsy positive OSCC patients aged 30–71 years of either gender were recruited from the Oral and Maxillofacial Surgery Department, Mayo Hospital, Lahore, Pakistan from July to December, 2016 with prior informed consent. Non-probability purposive sampling technique was used. Patients receiving chemotherapy/radiotherapy or having rheumatoid arthritis, diabetic foot or acute myocardial infarction were excluded. Age and gender matched thirty-eight healthy subjects aged from 30 to 70 years of either gender were also included as control group. Demographic and clinical findings of both the groups were recorded in the prescribed proforma. Ethical approval of the study was obtained from the Institutional Review Board of King Edward Medical University Lahore Pakistan (KEMU).

Five ml venous blood sample from each subject of two groups was drawn in serum separator tubes (Vacutainer SST, Becton-Dickinson, USA), allowed to clot for 10-15 minutes and then centrifuged at 1700 g for four minutes. Clear serum supernatant thus obtained was separated in labeled microfuge tubes and stored at -80 ±5°C. Before assay the serums were thawed and brought to room temperature till the estimation of serum MMPs.

MMP 1-13 levels were measured by multiples ELISA using Bio-Plex Pro™ Human MMP Panel kit (9-Plex #171-AM001M, Bio-Rad, USA). The assay reactions were performed as per the instructions of the manufacturer and results read on Multi-Analyte Bio-Plex® MAGPIX™ Multiplex Reader (Bio-Rad, USA).

### Statistical Analysis

Statistical analysis was performed by MedCalc v15.8 statistical software. The difference of age, gender, smoking and substance-use between OSCC group and control group was analyzed by student t test and chi square test of independence. MMP levels were expressed as median and Interquartile Range (IQR). Mann-Whitney U test was used to compare the MMP levels between two groups. Statistical significance was assumed at P ≤ 0.05. Receiver Operating Characteristic (ROC) curve analysis was performed to determine the area under the curve (AUC) with 95% confidence interval (95% CI) for sensitivity and specificity of the statistically significant MMPs of both groups to evaluate the predictive value of OSCC.

## RESULTS

There was no statistical difference in terms of age, gender, smoking and substance use in the two groups (Table-I). Of the twenty seven smokers, 96% (n =26) were males and 4% (n = 1) were female (4%). Among substance users (n = 40), twenty seven (67.5 %) were males and thirteen (32.5) were females. However, 55% (n = 22) used betel leaf/ tobacco /nuts, 37.5% (n = 15) were tobacco sniffers while 7.5% were mix users.

**Table-I:** Demographic Characteristics.

Characteristic	Type	Patients (n = 38)	Control (n = 38)	P value ^a^
Age (year)^c^		50.9 ± 9.4	50.8 ± 10.3	0.952
Gender (n)	Male	26	23	0.472^b^
	Female	12	15	
Smoking (n)	Yes	14	13	0.810^b^
	No	24	25	
Substance use (n)	Yes	21	19	0.645^b^
	No	17	19	
Lesion Differentiation	Well	20		
	Moderate	16		
	Poor	03		

a p value calculated by t-test or chi square;

b statistical significance; p < 0.05;

c Mean ± SD

The OSCC lesion was well differentiated in 51% (n= 20), moderately differentiated in 41% (n= 16) and poorly differentiated in 8% (n= 2). The predominant site of OSCC lesion was tongue (40%).

Median and Interquartile range (IQR) were calculated for each of the nine MMP levels (pg/ml) for OSCC and control groups. The plasma levels of MMP-1 (p = 0.002), MMP-8 (p = 0.001), MMP-10 (p = 0.001), MMP-12 (p = 0.002) and MMP-13 (p = 0.006) showed statistically significant increase in OSCC patients as compared to controls. The values of serum MMPs in OSCC patients and healthy subjects are given Table-II.

**Table-II:** MMP Levels (pg/ml) in OSCC and Control Groups.

MMP Type	OSCC Group Median (IQR)	Control Group Median (IQR)	P value^a^
MMP-1	565.8 (325.0 to1199.9)	350.5 (258.0 to 740.1)	0.002
MMP-2	2345.9(1861.2 to 3686.0)	4006.4(2569.1 to 6317.6)	0.002
MMP-3	858.3(313.8 to 1713.3)	1038.7(447.4 to 1879.0)	0.55
MMP-7	263.9(165.6 to 468.4)	173.9(122.0 to 338.2)	0.13
MMP-8	927.5(421.7 to 2219.1)	325.4(152.3 to 911.8)	0.001
MMP-9	6491.8(2794.4 to 11004.8)	4138.6(2213.0 to 12829.4)	0.42
MMP-10	77.0(21.9 to 368.8)	27.0 (12.2 to 65.4)	0.001
MMP-12	47.2(18.6 to 141.0)	10.7(6.7 to 16.1)	0.002
MMP-13	5.0(1.5 to 9.1)	1.8(1.5 to 3.2)	0.006

a Mann-Whitney U-test, p ≤ 0.05 was considered statistically significant.

**Fig.1:**
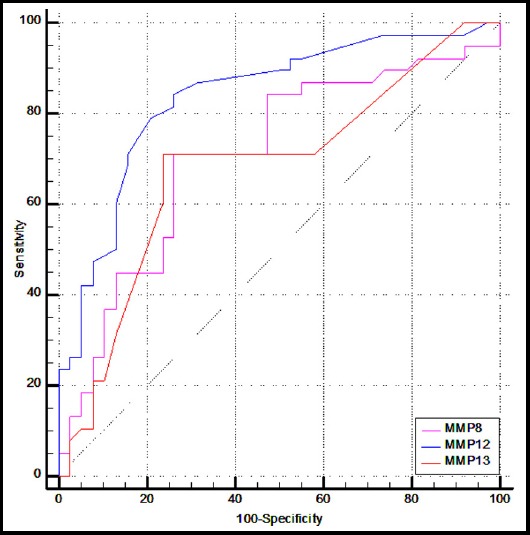
ROC Curve Analysis of MMPs

ROC curve analysis showed that almost all the MMPs had AUC more than 0.500 (Table-III). Among the MMPs having statistically significant increase in their values for OSCC group as compared to control group, the MMP-12 predicted the presence of OSCC with highest AUC of 0.836 (95% CI [0.733 to 0.911]) for sensitivity and specificity of 80.0% and 78.9%, respectively (Fig.1).

**Table-III:** ROC Curve Analysis for MMPs.

Marker	ROC parameters				

	AUC	95% CI	Sensitivity (%)	Specificity (%)	Cut- off value (pg/ml)
MMP-1	0.642	0.524 to 0.749	71.1	55.3	>362
MMP-2	0.704	0.588 to 0.803	65.8	73.7	≤2726.15
MMP-3	0.539	0.421-0.655	28.9	86.8	≤313.8
MMP-7	0.600	0.482-0.711	73.7	55.3	>183.91
MMP-8	0.706	0.591 to 0.805	71.1	73.7	>659.93
MMP-9	0.554	0.436-0.668	55.3	65.8	>5929.88
MMP-10	0.721	0.606 to 0.817	36.8	100	>160.45
MMP-12	0.836	0.733 to 0.911	80.0	78.9	>16.13
MMP-13	0.681	0.564-0.783	71.1	76.3	>3.24

## DISCUSSION

Histologically, squamous type OSCC is the most prevalent cancer of oral cavity causing about 85–90% cancer burden in Pakistani population.^11^ The pursuit of a biomarker for the timely and precise detection of OSCC is ever increasing. However, different biomarkers have been tested and remained in use despite different limitations.

In this study we measured levels of 9 MMPs in age and gender matched OSCC patients and controls. Individually except MMP-3, -7 and -9 all others showed difference in their median serum levels between the two groups. However, the median serum level of MMP-2 was statistically higher in controls as compared to OSCC patients thereby giving idea about some feedback regulation.

The role of higher MMP-2 expression was found linked to metastatic potential and poor survival in OSCC patients as observed in archival tissue immunohistochemistry of 30 OSCC patients and 10 controls. The expression of MMP transcripts was not measured by qPCR and did not measure the plasma levels.^12^ Another study reported that the immunohistochemical expression analysis of MMP-2 supported the prognostic aggressiveness of head and neck tumors,^13^ however no measurement of plasma levels found. A study by Kamata et al. showed that cell invasion drastically increased with elevated expression of MMP-2.^14^ However, our results regarding MMP-2 seem to be different, whereby the plasma levels were not increased, from previous researches which reported increased in expression. Zhang et al. showed higher levels of MMP-1 in OSCC associated fibroblasts when compared to normal fibroblasts.^15^ Similarly, Ha et al. linked invasiveness of OSCC to enhanced levels of MMP-1 and -10.^16^

A study by Ahmad et al. on MMP-7, -8 and -9 in oral and cutaneous squamous cell cancers showed MMP-7 expression to be elevated at the invasion front and MMP-9 mildly expressed. However, contrary to our results MMP-8 was not found in the OSCC but was present, along with MMP 9, in peri-cancerous inflammatory cells.^17^ A study by Korpi et al. stated a positive association of MMP-8 with patient survival thus hypothesizing a protective role of MMP-8.^18^ In accordance with our findings, Stott-Miller et al. observed highly elevated, yet not statistically significant, levels of MMP-1 and -3 in saliva of patients with OSCC compared to healthy controls.^19^

Makinen et al. associated MMP-13 with invasion depth and poor prognosis.^20^ Of all the nine MMPs under investigation in the current study, a single proteinase (MMP-12) was identified with high confidence (p< 0.05) and 4 proteinases (MMP-2, -8, -10 and -12) showed a significant expression change between cancer patients and healthy controls.

Yen et al reported MMP-1 and MMP-10 as potential oral cancer marker by using AUCs of relative gene expression as 0.715, 0.727 and 0.513 for MMP-1, MMP-10 and MMP-12, respectively.^21^ While in our findings, MMP-12 predicted the presence of OSCC with highest AUC of 0.836 (95% CI [0.733 to 0.911]) for sensitivity and specificity of 80.0% and 78.9%, respectively in serum.

Using ROC curve analysis, we have shown for the first time in Pakistan that a biomarker panel measuring serum MMPs has the ability of reliably differentiating normal healthy individuals from patients of biopsy positive OSCC. These findings suggest that this novel biomarker has great potential for clinical use in both diagnosis and management.

### Limitations of the study and Future Directions

A smaller sample size from a single hospital was used. A larger and diverse sample along with the evaluation of genetic expression of each MMP would provide better insight for diagnostic, prognostic and therapeutic roles.

## CONCLUSION

It is clearly seen from the current data that out of all the MMPs used in this study, only MMP-12 has been found to have significant sensitivity and specificity to be recommended as a diagnostic biomarker.

### Authors Contribution

**NC:** Conceived, designed and final approval of manuscript.

**SS & NAW:** Data collection, performance of tests and manuscript writing.

**AJG:** Statistical analysis, review and editing of manuscript.
